# A Gradient Microstructure Improves the Barrier Properties of Flake-Filled Composite Films: A Computational Study

**DOI:** 10.3390/ma16041691

**Published:** 2023-02-17

**Authors:** Thanasis D. Papathanasiou, Michalis Diakonikolis, Andreas Tsiantis

**Affiliations:** Department of Mechanical Engineering, University of Thessaly, 38334 Volos, Greece

**Keywords:** barrier properties, flakes, gradient composite materials, diffusion, microstructure

## Abstract

Composite films of a graded miscrostructure hold the promise of achieving optimal use of the filler material, resulting in composites with improved and application-taylored properties. In the context of barrier materials in which the reinforcing phase comes in the form of flakes or platellets, concentrating the filler particles in certain critical regions is thought to achieve economy in filler usage while ensuring superior barrier performance. The objective of the present article is to quantitatively test this hypothesis and provide guidelines on the expected barrier improvement. A model is developed, according to which a graded miscostructure in a composite film offers a quantitative improvement over an equivalent homogeneous microstructure; this improvement is quantified using a coefficient β, which depends on the form of the graded miscrostructure, specifically the distribution of the number-density of the filler particles across the film. It is shown that β=1 for a uniform microstructure and β>1 for a graded one, indicating that a graded miscrostructure will indeed result in improved barrier properties. Analytical expressions for β are developed for certain typical distributions; for a linear filler distribution, it is shown that β=4/3. This model is tested against detailed multi-particle simulations and is found to be in excellent agreement with computational results.

## 1. Introduction

Materials of a gradient microstructure, otherwise known as functionally gradient materials (FGMs), form a class of heterogeneous materials that exhibit a gradual transition of composition or morphology between two extreme states [1]. One example of compositional grading is seen in metal–matrix composite FMGs used in coatings or components designed to afford thermal or wear protection as well as minimize the effect of thermal stresses or the propagation of cracks; in these, a ceramic-rich exterior is seamlessly connected to a metallic interior by a material having a graded composition [2]. An example of microstructural grading can be found in the case of articular cartilage, a specialized connective tissue found in synovial joints. In this, the orientation of the constituent collagen fibers changes gradually from parallel to perpendicular to the articular surface [3,4], resulting in an optimal distribution of stress. FGMs abound in nature, as, for example, in the structures forming human bone, in several sea-shell tissues as well as in plants. The earliest reference to manufactured FGMs appeared in the late 1980s with the pioneering work of Japanese researchers [5,6] in the context of the development of thermal protection materials for aerospace applications. Since then, many applications of FGMs have appeared in bio-medical devices (prosthetics, for example, in the form of various HAP/Ti composite coatings [7,8,9]), nuclear energy, and other high-temperature applications [10,11], sensors and actuators, electricity generation, optical components, and many others. The advent of additive manufacturing and the flexibility it offers to gradually vary the composition or morphology in a component at the microstructure level has resulted in a renewed interest in FMGs in recent years [12], while a recent review of FGMs as related to manufacturing and properties can be found in [13]. In the area of micro-scale heat transfer, the effect of a graded microstructure has been studied, computationally and experimentally, by Dondero and co-workers [14]; the methodology, both computationally (the use of the fast multiple boundary element method, e.g., [15]) and experimentally (holes drilled in an aluminum plate, thus obtaining a variety of gradient profiles) can be extended to construct and analyze more general model systems of the gradient microstructure.

Contrary to the above applications, the concept of a gradient structure has not yet been fully utilized in the area of flake composites, even though multi-layer applications involving flake composites appear, for example, in sound insulation using mica flakes [16]. Flake composites, in which the reinforcing particles have the form of platelets (flakes), are characterized by a high aspect ratio and the very high area-to-volume ratio of the reinforcing particles. In mass-transfer applications, these particles act as local impermeable barriers and, therefore, force diffusing solute molecules to follow a highly tortuous path as they diffuse through the material [17]. This results in significantly reduced effective diffusion coefficients, and thus flake composites have found usage in numerous applications in which the diffusion rate of gasses through a film needs to be minimized, such as in corrosion [18], fire resistance [19], and packaging [20]. The prediction of the effective diffusion coefficient $D_{eff}$ in such composites is well advanced [21,22,23,24,25,26,27,28,29], as in the case when the flakes are uniformly distributed in the composite. However, the potential effect of a graded distribution of the flakes on their barrier effectiveness has not been studied. The objective of this study is to fill this gap and provide estimates for the barrier improvement factor ($BIF=D_{0}/D_{eff}$) of gradient flake composites for the base-line case of flakes aligned normally to the direction of macroscopic diffusion. To our knowledge, this is the first study that deals with flake composites of a graded structure. The paper is organized as follows: In Section 2, we develop a novel closed-form model for the BIF of composites having various common graded structures. In Section 3, a detailed computational analysis of the relevant systems is presented. Section 4 summarizes the computational results and compares them to the predictions of the analytical model.

## 2. A Model for the Effective Barrier Improvement Factor (BIF) of a Graded Composite Film

**Figure 1:**
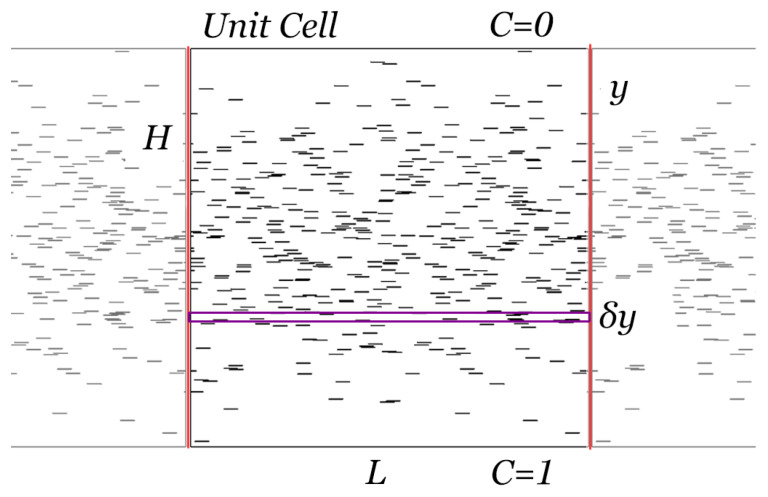
Schematic of a 2D unit cell, as used in this work, of height *H* and width *L*. Additionally, the boundary conditions on the top and bottom surfaces are shown. The geometry is periodic in the horizontal direction, and periodic boundary conditions are applied on the vertical boundaries of the unit cell. In this example, $\bar{\alpha \phi }$ = 0.5 where $\bar{\alpha \phi }$ is the macroscopic average flake concentration. In total, 500 flakes were placed in the unit cell so that their vertical positions followed a normal distribution with maximum density at 0.5*H* and σ = 0.2.

Consider a distribution of flakes, as shown in the schematic of Figure 1, in which N(y) is the local density of the flakes at a location y of height δy. This is related to the total number of flakes across the unit cell through $N_{T}=\int_{0}^{H}N\left(y\right)dy$. At a location *y*, the local concentration measure will be αϕ(y), where ϕ is the local volume fraction of flakes in a horizontal cross section and α is the flake aspect ratio, defined as the ratio between flake length *l* and flake thickness *t*. It can be shown [29] that the product αϕ can be expressed as $\alpha \phi \left(y\right)={\left(l/H\right)}^{2}N\left(y\right)$. Using this, the local BIF, if expressed by the model of Lappe et al. [25], will be

(1) $$BIF\left(y\right)=\frac{D_{0}}{D_{eff}}={\left(1+\frac{\alpha \phi (y)}{\lambda }\right)}^{2}$$

where $D_{0}$ is the diffusion coefficient of the solute molecules in the matrix material; $D_{eff}$ is the effective diffusion coefficient of the same molecules in the composite; and λ is a geometrical factor, expressing the (increased) path a solute molecule is forced to take as it navigates its way around a flake. The model of Lappe at al. [25] has been found to be in excellent agreement with the results of 2D and 3D computations in spatially uniform composites and, for 2D systems, the geometrical constant λ has been found to take the value of λ=2.5 [30,31]. We will use this value in this study. In light of Equation (1), an average value of the BIF across the film will be

(2) $$\bar{BIF}=\frac{1}{H}\int_{0}^{H}{\left(1+\frac{\alpha \phi (y)}{\lambda }\right)}^{2}dy$$

Equation (2) is based on the assumption that mass transfer across the gradient composite can be described by a resistance-in-series model. This is a correct assumption for layered composites containing randomly placed flakes, in which no low resistance paths form through flake layers. Expanding the right-hand-side of Equation (2), we obtain

(3) $$\bar{BIF}=1+\frac{2}{\lambda }{\left(\frac{l}{H}\right)}^{2}N_{T}+\frac{\lambda^{4}}{\lambda^{2}H^{3}}\int_{0}^{H}N{\left(y\right)}^{2}dy$$

Obviously, $N_{T}=\bar{\alpha \phi }{\left(H/l\right)}^{2}$ and therefore the second term in the right-hand side of Equation (3) is $\left(2/\lambda \right)\left(\bar{\alpha \phi }\right)$. The third term in the right hand side of Equation (3) can be written as

(4) $$\frac{N_{T}^{2}l^{4}}{\lambda^{2}H^{4}}\times \frac{H}{N_{T}^{2}}\int_{0}^{H}N{\left(y\right)}^{2}dy={\left(\frac{\bar{\alpha \phi }}{\lambda }\right)}^{2}\times \beta$$

where

(5) $$\beta =\frac{H}{\left(N_{T}^{2}\right)}\int_{0}^{H}N{\left(y\right)}^{2}dy$$

is a positive factor that depends only on the form of the distribution N(y); evidently, β expresses an improvement in barrier effectiveness brought about by the graded distribution of the flakes. Through Equations (3) and (4), the effective BIF can be expressed as

(6) $$\bar{BIF}=1+\frac{2}{\lambda }\times \bar{\alpha \phi }+\beta \times {\left(\frac{\bar{\alpha \phi }}{\lambda }\right)}^{2}$$

According to Equation (6), the BIF of a graded composite film is expected to be higher than the BIF of an equivalent (same number and dimensions of flakes per unit area/volume) composite in which there is no graded structure, but, instead, the flakes are uniformly distributed throughout the film thickness. Obviously, in the latter case, it can easily be shown that $\alpha \phi \left(y\right)=\bar{\alpha \phi }$ and β=1. Notice that, from Equation (6), it is clear that β operates on the quadratic term of the BIF model; therefore, its effect will be seen best in concentrated systems. Conversely, grading is only expected to have a minor effect in dilute flake dispersions since, in such systems, BIF is dominated by the linear term in ($\bar{\alpha \phi }$).

Equation (6) is significant since, if verified, it provides a direct means of assessing the increase in barrier effectiveness caused by grading and helps to identify microstructures that offer the best barrier enhancement. In the following, we will perform computer simulations in order to determine the range of validity of Equation (6). We will consider the following four types of flake distributions:

(i) A half-normal distribution, according to which the flake concentration is maximum on the surface (y=1) and minimum at the bottom (y=0) of the film.
  In this case, the number-density of the flakes N(y) is given by
  (7) $$N\left(y\right)=\frac{A}{\sigma }\sqrt{\frac{2}{\pi }}\exp\left(-\frac{y^{2}}{2\sigma^{2}}\right)$$
  where the constant A is related to the total number of flakes $N_{T}$ through a total balance: $N_{T}=\int_{0}^{H}N\left(y\right)dy$. From Equation (5), it can be shown that in this case the enhancement factor β is a function of the steepness of the distribution (expressed by the parameter σ). The relation between β and σ is:
  (8) $$\beta =\beta \left(\sigma \right)=\frac{erf\left(\frac{1}{\sigma }\right)}{\sigma \sqrt{\pi }\times erf{\left(\frac{1}{\sigma \sqrt{2}}\right)}^{2}}$$
  It can be formally shown that as σ→∞, then β→1. This result has also been verified with numerical computations, as will be shown later in this paper.
(ii) A linear distribution between the two sides of the film. In this case, it is N(y)=ky, where *k* is a constant. Since $N_{T}=\int_{0}^{H}kydy$, it follows that $k=2N_{T}/H^{2}$, and therefore $N\left(y\right)=\left(2N_{T}/H^{2}\right)\times y$. Taking into account that $\alpha \phi \left(y\right)={\left(l/H\right)}^{2}N\left(y\right)$ and introducing this in Equation (5), we derive
  (9) β=4/3
(iii) A triangular distribution in which the flake concentration is maximum at the center and drops linearly towards the two edges of the film. It can be shown that in this case it is also β=4/3.
(iv) A normal distribution, with the maximum flake density occurring at the center of the film (y=0.5H). In this case, the number-density of the flakes varies as
  (10) $$N\left(y\right)=\frac{A}{\sigma \sqrt{2\pi }}\exp\left[-\frac{1}{2}{\left(\frac{y-0.5H}{\sigma }\right)}^{2}\right]$$
  In a manner similar to the one used in the case of the half-normal distribution, the enhancement factor β can be shown to depend only on the variance (σ) of the distribution; the result is:
  (11) $$\beta =\frac{erf\left(\frac{0.5H}{\sigma }\right)-erf\left(-\frac{0.5H}{\sigma }\right)}{\sigma \sqrt{\pi }\times {\left(erf\left(\frac{0.5H}{\sigma \sqrt{2}}\right)-erf\left(-\frac{0.5H}{\sigma \sqrt{2}}\right)\right)}^{2}}$$
  It can be shown that as the variance of the distribution increases, the enhancement factor β approaches 1 asymptotically.

## 3. Computational

**Figure 2:**
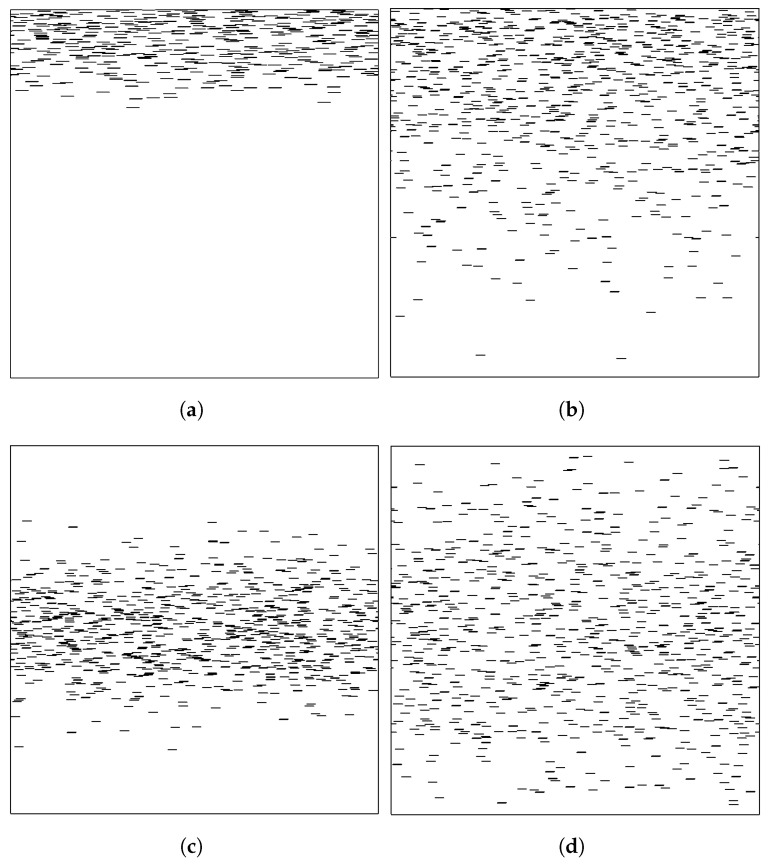
Typical unit cells with various flake distributions. As σ increases the distribution of flakes approaches a random placement. In all of the above images, *N* = 800 and $\bar{\alpha \phi }=0.5$. (**a**) Half normal distribution with σ=0.1. (**b**) Half normal distribution with σ=0.3. (**c**) Normal distribution with σ=0.1 and maximum at the middle of the Unit Cell. (**d**) Triangular distribution.

Computations are carried out in two-dimensional unit cells of square shape (side length H), which are formed by placing $N_{T}$ flakes according to a desired distribution N(y). The flakes are unidirectional, with their plane perpendicular to the direction of the macroscopic diffusion (y). We are interested in the case of thin flakes, whose cross-sections appear as 1D lines, parallel to each other and perpendicular to the direction of the macroscopic diffusion, as shown in Figure 2. When the flakes are aligned with their axes perpendicular to the direction of diffusion, the hindrance to diffusion (and thus the BIF) are maximized. For this reason, this form of flake alignment is the preferred alignment in flake composites and is the configuration mostly studied, computationally as well as theoretically. Most resin processing/application methods for polymer melts, epoxy resins, or paints involve flows with substantial elongational or shear components. These flows are known to align the flakes in the direction of flow [32], which in practical applications is the same as the direction of the coated substrate and normal to the direction of macroscopic diffusion.

**Figure 3:**
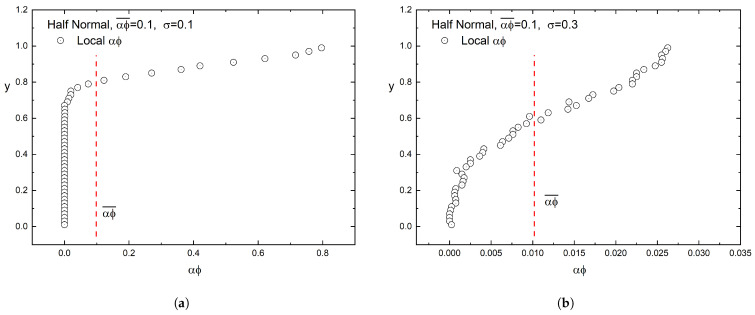
Typical flake concentration profiles ranging from a steep concentration gradient to a more uniform distribution of flakes. (**a**) σ=0.1, (**b**) σ=0.4. In both cases, $\bar{\alpha \phi }=0.1$.

The state of a flake dispersion is characterized as dilute, semi-concentrated, or concentrated, based on the value of the product αϕ, where α=l/t is the flake aspect ratio and ϕ is the flake volume fraction. If αϕ>1, we are in the concentrated regime. If 1/α<αϕ<1, we are in the semi-concentrated regime, while a dispersion in which αϕ<1/α is considered dilute. Additionally, since in our case the placement of flakes is non-uniform, the local αϕ varies, and at various positions it can exceed the above characterization limits. This is especially evident at smaller values of σ since these lead to more dense areas inside the unit cell due to higher concentration of flakes. So, even if a film is characterized by an average value of αϕ, locally it shows a different character, and this affects the overall barrier performance. This is ilustrated in Figure 3.

**Figure 4:**
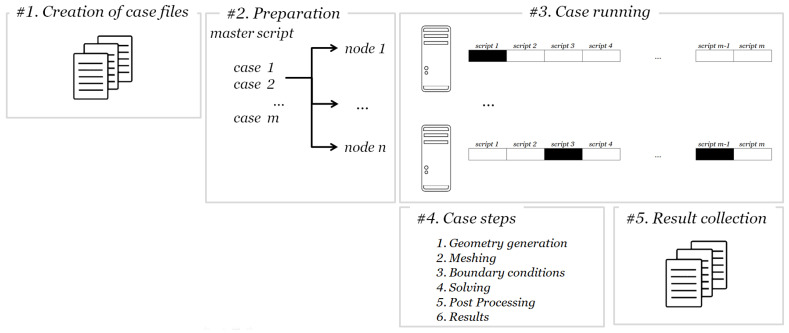
Flow chart of the simulation setup. In step #1, the geometry data of the flake positions and unit cell is created using in-house application codes. The applications create (a) the appropriate case files, (b) the master script that runs in the main cluster node, and (c) the individual case sub-scripts. In step #2, the case files are uploaded to the cluster and the master script is executed at a master node sending the individual scripts to the computational nodes. Each node runs the cases in step #3 using a greedy algorithm. Every script that runs (step #4) creates the geometry using boundary representation geometry and then creates the mesh. Using OpenFOAM utilities, the mesh is converted, boundary conditions are applied, and the discretized model is partitioned according to the physical cpu cores in every node and executed in parallel. Finally, the results are obtained using again the OpenFOAM utilities. At step #5, the results are collected using in-house utilities for further analysis.

As shown in Figure 4, the procedure that is used for running simulations for each set of geometrical parameters consists of the following steps:

1. A set of parameters is determined, and the in-house application generates the appropriate geometry files. A unit cell is created with the desired dimensions, and for each set of ϕ and distribution the number of flakes is calculated. Then, the flakes are placed using a random sequential addition algorithm [33], according to which random numbers define the coordinates of the center of each line. The RSA algorithm performs checks to ensure no flake overlap occurs, and to ensure that a minimum separation distance between neighboring flakes is maintained. The latter is essential so that a computational mesh can be generated in the narrow gaps separating adjacent flakes. After the completion of the unit cell calculation, the case directories are created with the appropriate scripts for execution. A master script is created that will run in the main node and coordinate the execution at the various nodes.
2. The master script is executed; it uses a greedy strategy to assign the cases to each computational node. According to this strategy, each node in the computational cluster sequentially scans the case directories until an ‘unsolved’ case is found. In this case, the actions in step 3 are executed. If all of the cases are scanned and no other ‘unsolved’ cases are found, the script terminates its execution.
3. The nodes are searching for the existence of status files inside each directory that indicate the state of the case. If the status is ‘unsolved’, it is flagged as ‘running’ and the node starts the execution of a sub-script that runs the simulation. If a different node accesses that case folder, it will read the status and will skip it if its status is any other than ‘unsolved’. If a case is ‘unsolved’, then the actions of step 4 are executed.
4. A sub script is run in each case directory that executes the commands for running the simulation. The first step is the creation of the mesh. Then the mesh is converted using OpenFOAM utilities, and the boundary conditions are applied. The flakes are being simulated as being one dimensional entities that have only length or ‘baffles’, as referenced in the boundary conditions settings. In the case of baffles, boundary conditions are applied to the internal faces of both cells that lie on each side of the geometrical entity, effectively creating an internal boundary inside the mesh. The simulation is split in order to run in parallel using the the public domain openMPI implementation of the standard message passing interface (MPI). After a solution is achieved, the results are written in a file and the case is flagged as ‘solved’.
5. The in-house application is used to scan the case directories and is retrieving the simulation results. Here, it is interesting to note that there is no need to finish all of the simulations before the results are obtained. The results that have been obtained at any time can be collected as needed.

These simulations were run in the Linux environment of the National HPC facility ARIS using 8 nodes with 16 cpus each.

The unit cells are periodic in the x-direction. The boundary conditions for concentration are cyclic in the x-direction, namely, C(0,y)=C(L,y). In the y-direction, a fixed concentration gradient ΔC is imposed; therefore, fixed concentration values are prescribed on the top and bottom boundaries, simulating the steady-state case of a film placed as a barrier between two regions. The flakes are considered to be impermeable, and thus at every point on their surface we have dC/dn=0. At each level of $\bar{\alpha \phi }$, we generate 10 different geometries, which differ from one another only by the spatial placement of the flakes. The files for each case setup and the geometric information regarding the unit cells is created using in-house developed applications after setting the flake distribution characteristics and the rest of the case settings. The geometric files are imported in the mesh-generation application GMSH [34]; the generated meshes consist of around ∼10${}^{7}$ triangular elements. More information on mesh generation and spatial mesh convergence is included in the Appendix A. The case files created above are then imported into OpenFOAM [35] and the Laplace equation, namely,

(12) $$\nabla^{2}C=0$$

is solved using the well-tested solver LaplacianFoam. The adoption of Equation (12) implies that the matrix material is assumed to be homogeneous throughout, also including the regions adjacent to the flakes. This is a simplification for certain material/flake combinations as it is known that the presence of flakes may alter the conformation/crystallization and thus the properties of the matrix in their vicinity [36,37].

The solution to Equation (12) gives the value of dC/dn at each point on the top/bottom boundaries, from which the total mass flux is computed as

(13) $$J_{n}=-D_{0}\int_{0}^{H}\frac{\partial C}{\partial n}dx$$

Knowledge of the mass flux allows us to compute an effective diffusion coefficient of the composite film as

(14) $$D_{eff}=\frac{D_{0}}{\Delta C}\int_{0}^{H}\frac{\partial C}{\partial n}dx$$

**Figure 5:**
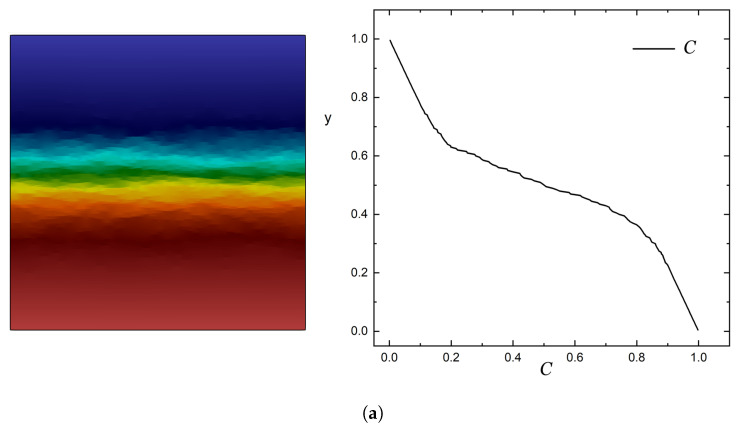

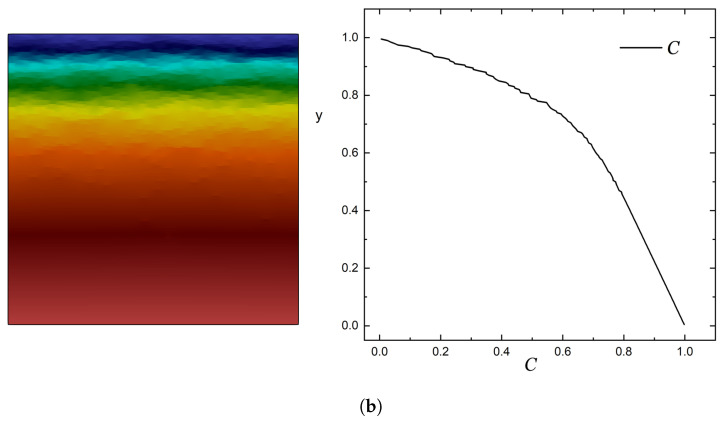
2D concentration maps (left) and concentration profiles along a vertical line across the center of the unit cell (right) for two typical cases. (**a**) Variation of concentration for a unit cell with $\bar{\alpha \phi }$ = 1.0 and flakes following normal distribution along the *Y* axis with the maximum at y=0.5H and σ=0.1. (**b**) Variation of concentration for a unit cell with $\bar{\alpha \phi }$ = 1.0 and flakes following half-normal distribution along the *Y* axis with maximum density at y=H and σ=0.2.

Example concentration profiles are shown in Figure 5.

## 4. Results and Discussion

### 4.1. Half-Normal Distribution of Flakes

**Figure 6:**
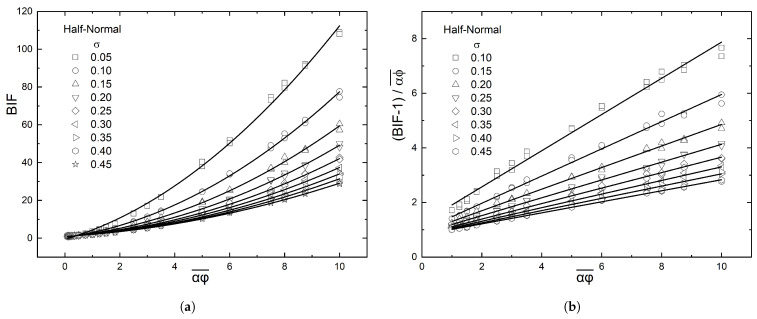
(**a**) Summary of results for the BIF of a composite film with a half-normal distribution of flakes. Each point represents the average of 10 simulations in geometries with different spatial distribution of flakes but the same $\bar{\alpha \phi }$ and N(y). The average flake concentration is on the horizontal axis; it is related to the total number of flakes $N_{T}=\bar{\alpha \phi }{\left(H/l\right)}^{2}$. (**b**) Presentation of the computational results as suggested by Equation (6); the slope of the line gives the value of the ratio $\beta /\lambda^{2}$.

**Figure 7:**
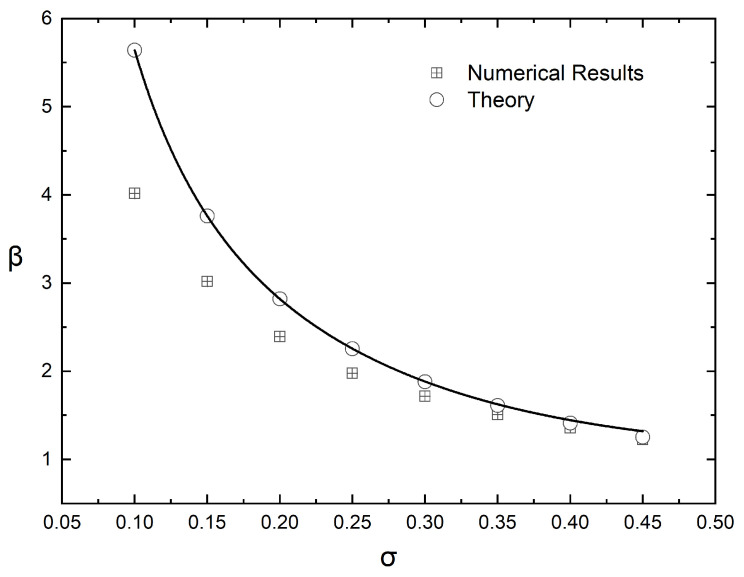
Variation of the enhancement factor (β) with the steepness of the distribution curve (σ) and its comparison to the predictions of Equation (8).

Equation (6) suggests that if the computed values of the BIF of the (graded) composite film are plotted as $\left(BIF-1\right)/\bar{\alpha \phi }$ vs. $\bar{\alpha \phi }$, they should fall on a straight line, whose slope will be equal to $\beta /\lambda^{2}$. This is indeed the case, as demonstrated in Figure 6, which shows that the enhancement factor (β) increases with increased grading (decreasing value of σ), and this increase is more evident for larger values of $\bar{\alpha \phi }$. The numerical results for β, obtained from Figure 6, are plotted in Figure 7 and also compared to the predictions of Equation (8).

**Figure 8:**
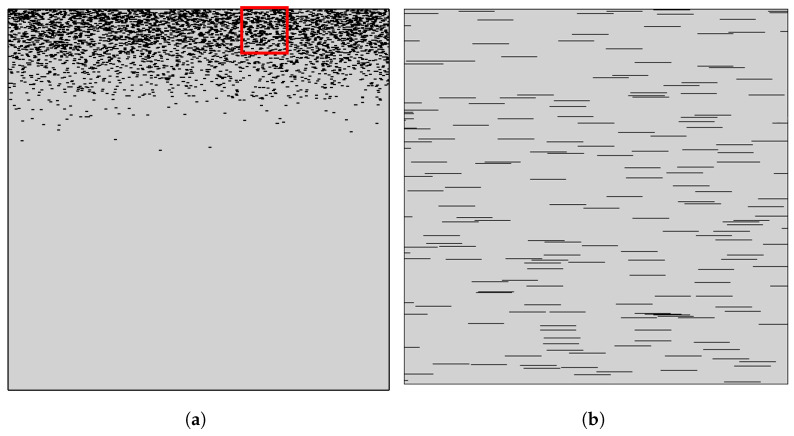
(**a**) Unit cell with N = 4000 and αϕ = 1.0. Flake placement follows a half normal distribution with σ=0.1. (**b**) Zoomed highlighted area as shown with a red outline in (**a**). We can see in the zoomed area that even in this dense configuration, there are flake-free pathways and flake stacking, which allow for faster diffusion at the local level.

It is clear that β asymptotically approaches the value of 1 with increasing σ− as expected since for σ→∞, the composite approaches a homogeneous state. It is also clear that the numerical results for β and the predictions of Equation (8) deviate at lower values of σ, that is, for films showing very high grading. The reason for this discrepancy can be found in the assumption underlying the derivation of Equation (6), namely, that the average BIF can be computed as a sum of resistances in a series. This assumption implies that there are no preferential (low resistance) paths in the flake layers. Such paths, however, can sometimes be found in very steep flake concentration profiles, in which case the great majority of the flakes are concentrated in a very thin subsurface layer, as can be seen in Figure 8.

### 4.2. Linear Distribution

**Figure 9:**
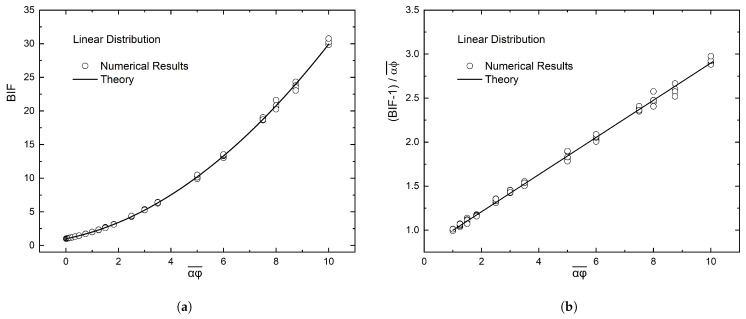
(**a**) Summary of results for the BIF of a composite film with a linear distribution of flakes, namely, N(y)=ky. In the horizontal axis is the average flake concentration, expressed as $N_{T}=\bar{\alpha \phi }{\left(H/l\right)}^{2}$. (**b**) Presentation of the computational results as suggested by Equation (6); the slope of the line gives the value of the ratio $\beta /\lambda^{2}$.

Equation (9) predicts that in the case of a linear variation of flake number-density, the enhancement factor is β=4/3. In this case, plotting the results for the BIF as in the previous section, namely, $\left(BIF^{-1}\right)/\bar{\alpha \phi }$ vs. $\bar{\alpha \phi }$, should result in one straight line, whose slope should be $\beta /\lambda^{2}=0.21$—if we take λ=2.5. Figure 9 shows the results, which form a straight line with slope 0.209, resulting in a value of β equal to 1.306, compared to a value of 4/3 = 1.333 expected from theory.

### 4.3. Triangular Linear Distribution

**Figure 10:**
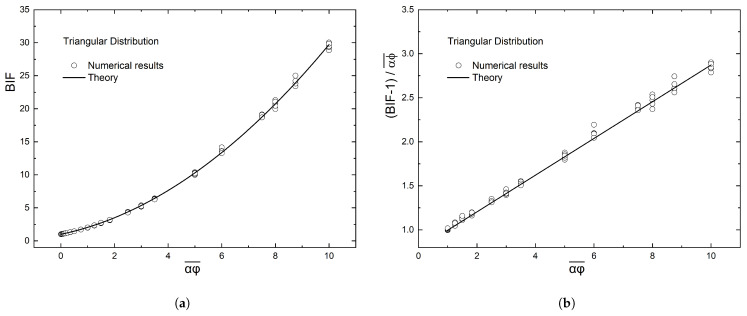
(**a**) Summary of results for the BIF of a composite film with a triangular linear distribution of flakes, having a maximum value at the center of the film. In the horizontal axis is the average flake concentration, expressed as $N_{T}=\bar{\alpha \phi }{\left(H/l\right)}^{2}$. (**b**) Presentation of the computational results as suggested by Equation (6); the slope of the line gives the value of the ratio $\beta /\lambda^{2}$.

As shown earlier, the value of β in this case should be 4/3. The computational results are shown in Figure 10. In this case, the slope of the regression line is 0.207.

**Figure 11:**
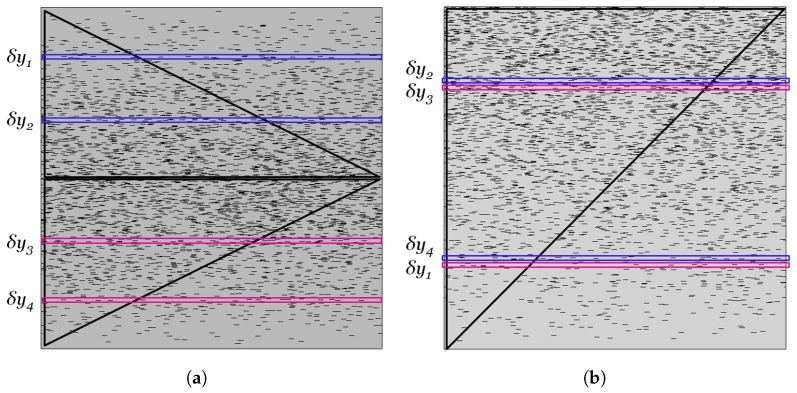
(**a**) Image of a unit cell with triangular distribution of flakes. The areas of height δy and flake number N(y) that were created from the distribution are shown. Also with black, the triangles that show the two areas of the triangular distribution around the maximum are superimposed on the unit cell image. (**b**) Image of a unit cell with linear distribution. The areas with the corresponding flake number N(y) from the first image are shown at their positions in the unit cell. It can be easily seen that geometrically the unit cell on the right can be produced from a linear combination of the areas δy from the unit cell in the left.

It is interesting to note here the similarities between the linear and triangular distribution. From the computational results, we see that they are virtually identical. This was to be expected, since these two distributions are geometrically similar. The linear distribution can be considered as a overlapping of two areas with triangular distributions. This is demonstrated in Figure 11, where we can see that a unit cell with linear distribution can be produced from a unit cell with triangular distribution.

### 4.4. Normal Distribution

**Figure 12:**
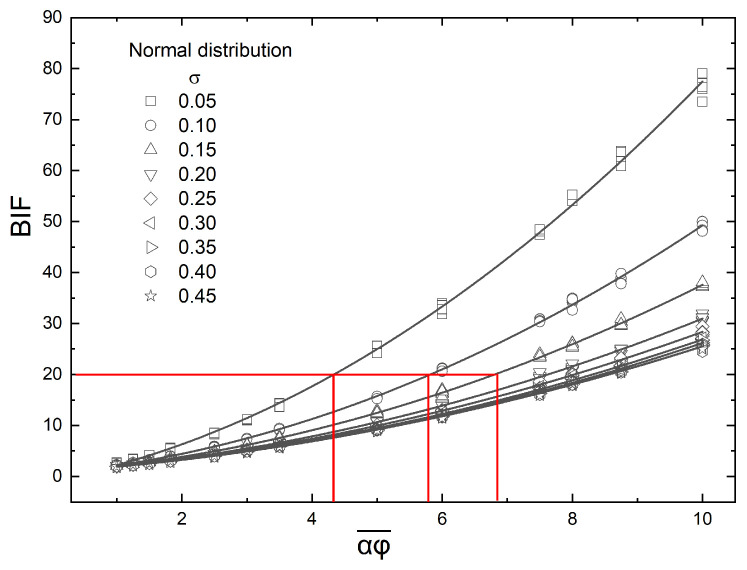
Summary of the computational results for the BIF of a composite film in which the flake concentration follows a normal distribution around the center of the film. The red lines show how the same BIF can be achieved from different $\bar{\alpha \phi }$ but with different distributions. It can be seen that flake distributions with smaller σ can achieve the same BIF with unit cells.

**Figure 13:**
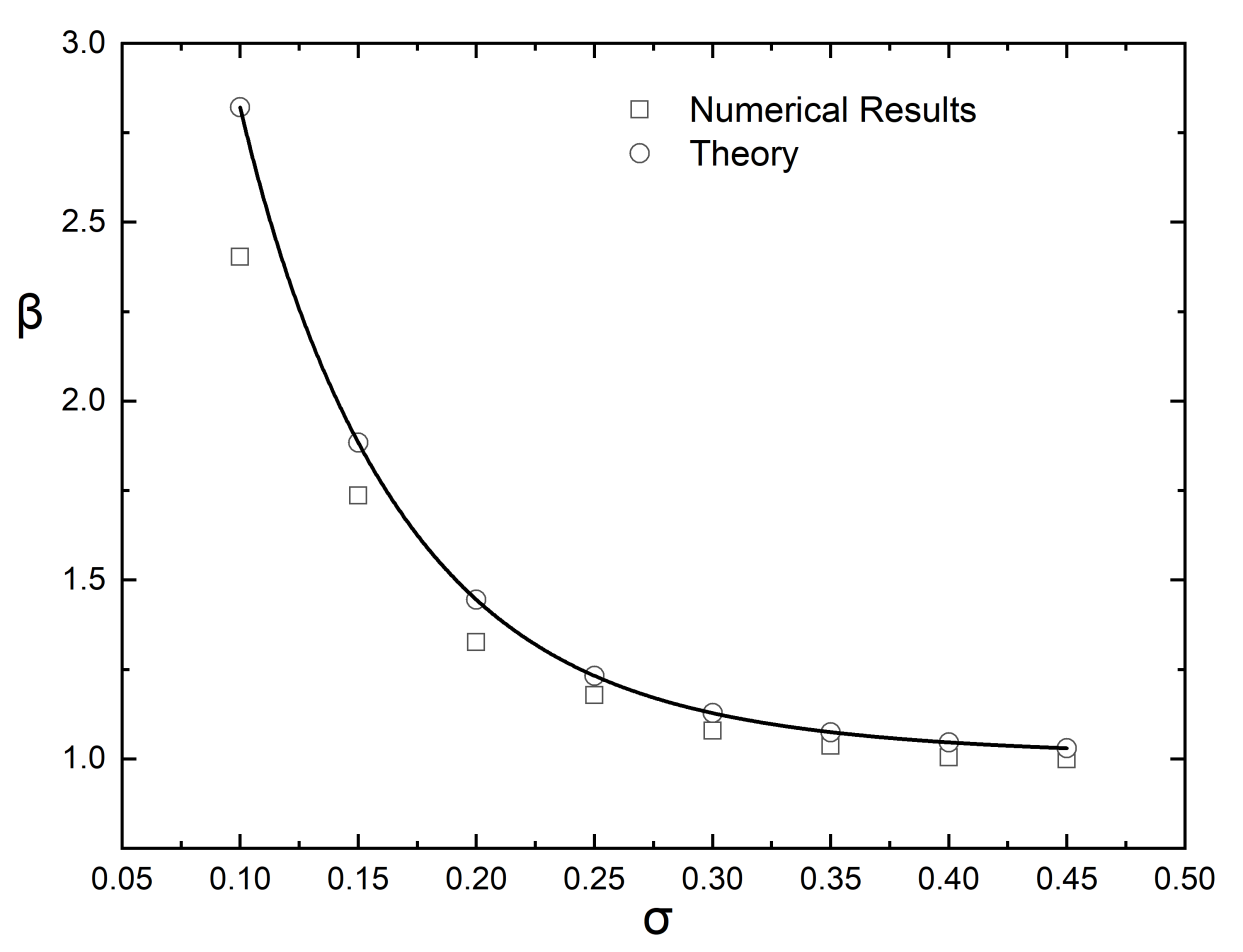
Comparison between computational results for β and predicted results from Equation (11).

Figure 12 is a summary of the results for the BIF in the case of a normal distribution of flakes, with the maximum concentration located at the center of the film as a function of $\bar{\alpha \phi }$ and σ. It can be seen that increasing σ beyond 0.3 results in very similar BIF vs. $\bar{\alpha \phi }$ curves; this is an indication that the gradient structure of the film will have an appreciable effect on its barrier properties only for relatively steep concentration gradients, typically σ<0.2. Figure 12 also provides a guide for film design; if a flake-filled film with a target BIF is to be obtained, this can be achieved using various total flake amounts, depending on the extent of grading. In the example of Figure 12, a BIF of 20 can be achieved with αϕ=4.3 if σ=0.05 (an extreme case of grading); this becomes 5.8 at σ=0.1 and 6.8 at σ=0.15. To achieve a BIF = 20 with uniformly distributed flakes, the required value of αϕ would have to be $\lambda (\sqrt{20}-1)=8.7$, which is double the flake amount if grading with σ=0.05 was used and 50% higher if a grading with σ=0.1 was used. The enhancement factor β was predicted to be a function of σ, as expressed by Equation (11). Figure 13 shows a comparison between the predictions of Equation (11) and the computational results for β.

## 5. Conclusions

Theoretical analysis and detailed computational results point to the conclusion that the barrier factor of a flake-filled film will increase if the spatial distribution of the flakes is non-uniform in the direction of macroscopic diffusion. The extent of improvement is directly linked to the form of the distribution through a coefficient (β) we introduce in this study. This coefficient operates on the quadratic term of the BIF model; therefore, the impact of grading is expected to be more significant in the semi-dilute and concentrated regimes. Analytical expressions for this coefficient are obtained for the cases of typical distributions such as linear, half-normal, and normal. From computational results, the coefficient β is also found and the obtained values are in very good agreement with the analytical expressions. Computational results also confirm that the highest impact of grading will be observed in concentrated films. Finally, our results are obtained for the case of fully-aligned flakes; for composites in which the flakes assume random orientations or in which the flakes are only partially aligned, the BIF reported here will form an upper bound [38].

**Figure 14:**
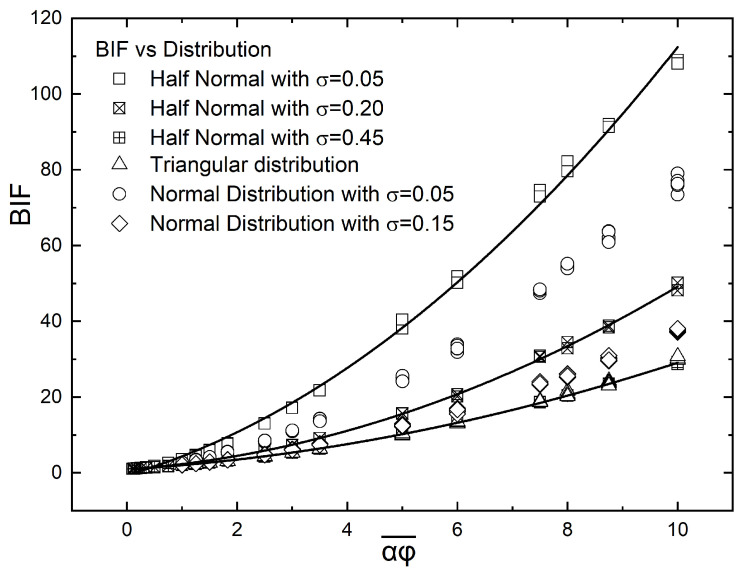
Comparison of the computed values of BIF for different distributions and the entire range of nominal αϕ. The linear distribution is not shown but its results are virtually identical with the triangular distribution. The effect of grading is most prominent in dense configurations and high degree of grading, with the half-normal distribution predicted to give the highest increase in BIF.

The values of BIF for different flake distributions and parameters are shown in Figure 14. We can see that the BIF values obtained from a half normal distribution with small σ are the highest. This is explained considering that the half normal distribution becomes very dense in the upper surface of the unit cell. This is also true for the normal distribution with σ=0.05 since this distribution also has a very dense area but this time in the middle of the unit cell. The triangular distribution and half triangular distribution (not shown in Figure 14 but giving almost identical results with the triangular distribution as shown in Figure 10) give the smallest increase in the BIF.

From the above it is concluded that in order to achieve high values of BIF, a manufacturing process which creates materials with highly flake-dense zones should be followed. If this is not achieved, then the BIF will effectively be the same as that obtained from a material with a random distribution of flakes. Small deviations from maximum grading can lead to a large reduction in BIF, as can be seen in the normal distribution where a difference from σ=0.05 to σ=0.15 can result in a two-fold reduction in BIF, making the barrier properties of the material similar to those of a material having a random distribution of flakes.

## Figures and Tables

**Figure A1:**
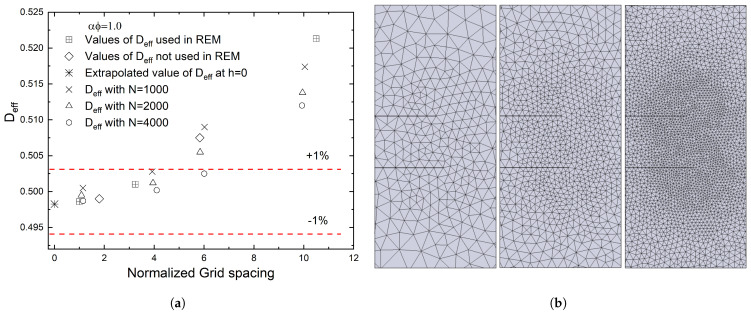
(**a**) An example of the application of the REM for a case with αϕ=1.0, including the datapoints used for the determination of the optimum number of flakes (*N*). REM was carried out for all simulation settings, and as a result it was concluded that meshes with around $10^{7}$ elements and flake number N=4000 are sufficient for all αϕ and σ levels examined. (**b**) Meshes created from the above procedure from coarse mesh (normalized grid spacing ∼10) to finer mesh (normalized grid spacing ∼1). A local size refinement was applied at the flake edges to more efficiently resolve the concentration gradients in these areas.

## Appendix A. Spatial Mesh Convergence

If a large number of demanding simulations are to be performed, as in the case of the present study, it is desirable to establish the grid size that will satisfy both the solution accuracy and the economy of the computational time. The Richardson extrapolation method (REM) [39] is a powerful tool in this regard and will be used in this section to confirm that the numerical results satisfy the criterion of spatial grid convergence. The REM allows us to obtain the highest order estimate for the value of an unknown function (*f*) as (a) the grid spacing approaches zero and (b) the number of elements (*N*) increases.

According to this method, the value of a function *f* obtained in a simulation is assumed to depend on grid spacing *h* and can thus be expressed as a series of *h*:

(A1) $$f=f_{h=0}+g_{1}h+g_{2}h+g_{3}h+...$$

where $g_{1}$, $g_{2}$, and $g_{3}$ are functions independent of grid spacing *h*, and $f_{h=0}$ is the value of the function *f* at zero grid spacing, that is, at an infinite number of elements (continuum value of *f*). Additionally, the quantity *f* is considered to be ‘second-order’ accurate if $g_{1}=0$. With the assumption of a second-order solution and by computing the quantity *f* on two grids of spacing $h_{1}$ and $h_{2}$, where $h_{1}<h_{2}$, an estimate of the value of *f* at zero grid spacing, if we neglect the third-order and higher terms, is:

(A2) $$f_{h=0}≅f_{1}+\frac{f_{1}-f_{2}}{r^{n}-1}$$

where *r* is the mesh refinement factor $r=h_{2}/h_{1}$. The continuum value $f_{h=0}$ is considered to be n+1 order accurate. Using the above, the order of convergence *n* is obtained as

(A3) $$n=ln\left(\frac{f_{3}-f_{2}}{f_{2}-f_{1}}\right)/ln\left(r\right)$$

For the purpose of this study, the function *f* is the effective diffusion coefficient $D_{eff}$ as calculated using Equation (14) and $f_{h=0}$ will be an estimate of $D_{eff}$ as the grid size approaches zero. $f_{1},f_{2}$ and $f_{3}$ are the calculated $D_{eff}$ values for three mesh sizes, which change from finer ($f_{1}$) to coarser ($f_{3}$). Since in our study we use three different values, the value $f_{h=0}$ will be calculated from

(A4) $$f_{h=0}≅f_{2}+\frac{\left(f_{1}-f_{2}\right)r^{n}}{r^{n}-1}$$

By calculating from Equation (A3), the rate of convergence *n* we can obtain an estimate for $f_{h=0}$ using Equation (A2) above. A similar approach can be followed using the number of flakes *N* instead of grid spacing *h*, with the difference being that instead of examining the number of flakes (*N*), we will examine the quantity 1/*N*, which approaches zero as *N* increases.

Besides determining the continuum value $f_{h=0}$, we can also estimate the quality of this result using the grid convergence index (B) [39] defined as

(A5) $$B_{fine}=B_{1,2}=\frac{S_{f}\left|e\right|}{r^{n}-1}$$

where $S_{f}$ is a safety factor whose values depend on the number of results (*f*) used; it is equal to 3 if 2 grids are used and 1.25 if 3 grids are used. The corresponding *B* of a coarser grid is calculated as

(A6) $$B_{coarse}=B_{2,3}=\frac{r^{n}S_{f}\left|e\right|}{r^{n}-1}=r^{n}B_{1,2}$$

If the ratio of the convergence indices $B_{1,2}/B_{2,3}$ from the above calculations has a value near unity, then the computed value for $f_{h=0}$ is inside the asymptotic range of convergence and is thus reliable.

An example of the application of the REM in our problem is given in the following, for a case with αϕ=1.0. For a given geometry, three different meshes are made using a refinement factor r=1.8. The results of the simulations are summarized in Figure A1.

In this typical case, the order of convergence is found using Equation (A3) and is equal to 3.6226. Using Equation (A4), the effective diffusivity ($D_{eff}$) is 0.49826. Since we used three grids with an effective ratio r=1.8, the $S_{f}$ is equal to 1.25. The ratio was kept constant by utilizing the setting of ‘CharacteristicMeshLength’ of GMSH. In order to calculate the asymptotic rate of convergence, we use Equations (A5) and (A6), and the resulting values are $B_{1,2}=0.08168$ and $B_{2,3}=0.68358$. The ratio $B_{2,3}/B_{1,2}=0.99518$∼1. Therefore, from the above analysis and comparing the results from the simulation with >$10^{7}$ grid elements, we can see that we are well within the asymptotic range. By performing a similar analysis for the number of flakes, we end up using *N* = 4000 for our simulations.
